# The gut microbiome and allograft outcomes: implications for heart transplantation

**DOI:** 10.1172/JCI207999

**Published:** 2026-08-17

**Authors:** Ivan Ðuran, W.H. Wilson Tang, Petra Mamic

**Affiliations:** 1 University of Zagreb School of Medicine, Zagreb, Croatia.; 2 Public Health Center (Dom Zdravlja) Zagreb-Centar, Zagreb, Croatia.; 3 Kaufman Center for Heart Failure Treatment and Recovery; Heart, Vascular and Thoracic Institute; and; 4 Department of Cardiovascular and Metabolic Sciences, Lerner Research Institute, Cleveland Clinic, Cleveland, Ohio, USA.; 5 Division of Cardiovascular Medicine, Department of Medicine;; 6 Department of Genetics; and; 7 Stanford Cardiovascular Institute, Stanford University School of Medicine, Stanford, California, USA.

## Abstract

Heart transplantation remains the gold standard therapy for patients with end-stage heart failure. However, post-transplant complications are considerable. Emerging evidence implicates the gut microbiome as a modifiable determinant of post–heart transplant outcomes through its influence on host immunity, metabolism, and inflammation. This Review synthesizes current understanding of gut microbiome dysregulation following solid organ transplantation, with particular emphasis on heart transplantation, examining mechanistic links underpinning important complications including allograft rejection, infection, metabolic dysfunction, and cardiac allograft vasculopathy. We critically evaluate bidirectional interactions between the gut microbiome and immunosuppressive drugs, assess the potential for microbiome profiling to serve as a predictive biomarker for post-transplant complications, and examine microbiome-targeted interventions including dietary modification, prebiotics, probiotics, and fecal microbiota transplant. Finally, we propose a translational roadmap to integrate microbiome science into heart transplant care to optimize immunosuppression, predict complications, and improve long-term outcomes for heart transplant recipients.

## Introduction

Heart transplant (HT) is a lifesaving intervention for patients with end-stage chronic heart failure (HF). Over 7,000 adult HTs are performed yearly worldwide, with over 4,000 performed in the United States (1, 2). Despite its transformative benefits, HT is frequently accompanied by serious complications, such as rejection, and requires lifelong immunosuppression, predisposing recipients to infectious, metabolic, and neoplastic complications.

Emerging evidence implicates the gut microbiome, a complex ecosystem of bacteria, viruses, fungi, and archaea residing within our gut, in modulating post-transplant physiology, complications, and outcomes. Understanding gut microbiome–host interactions following HT has the potential to enhance risk stratification, optimize current and enable novel therapies, and usher in personalized care strategies that would maximize benefit while minimizing toxicity for HT recipients. However, current microbiome-directed strategies remain investigational, with clinical translation requiring rigorous validation in adequately powered trials.

This state-of-the-art translational Review examines the gut microbiome’s role in HT. We begin by outlining major post-HT morbidity drivers and their pathophysiology, establishing clinical context for microbiome involvement. Following a concise overview of the gut microbiome fundamentals and its host crosstalk, we discuss microbiome relevance to HF and HT physiology and post-transplant complications. We summarize existing clinical data on microbiome alterations following heart and other solid organ transplants (SOTs) and explore the potential of leveraging the gut microbiome as a diagnostic, predictive, and therapeutic tool in HT. We conclude by identifying critical knowledge gaps and proposing actionable strategies to bridge discovery and clinical application to advance personalized care for future HT recipients.

## Allograft injury mechanisms and post-HT morbidity drivers

Heart allograft injury, one of the leading culprits of post-HT morbidity, occurs by multiple mechanisms, starting with ischemia/reperfusion injury (IRI). Blood flow cessation and restoration between organ procurement and implant incite sterile inflammation, endothelial injury, platelet adhesion, and complement activation (3). This initial tissue injury primes the immune system for subsequent alloimmune injury occurring via acute cellular or antibody-mediated rejection (ACR and AMR, respectively). In ACR, recipient T cells recognize donor HLAs, triggering recruitment of cytotoxic T cells, macrophages, and NK cells that attack allograft parenchyma and endothelium (4). In AMR, donor-specific antibodies (DSAs) target donor HLA on allograft endothelium to trigger complement-mediated endothelial activation, cytokine upregulation, macrophage infiltration, increased vascular permeability, and microvascular thrombosis (5). Cardiac allograft vasculopathy (CAV), a leading cause of late post-transplant morbidity and mortality (6, 7), is a form of chronic rejection causing diffuse coronary artery intimal hyperplasia.

**Figure 1:**
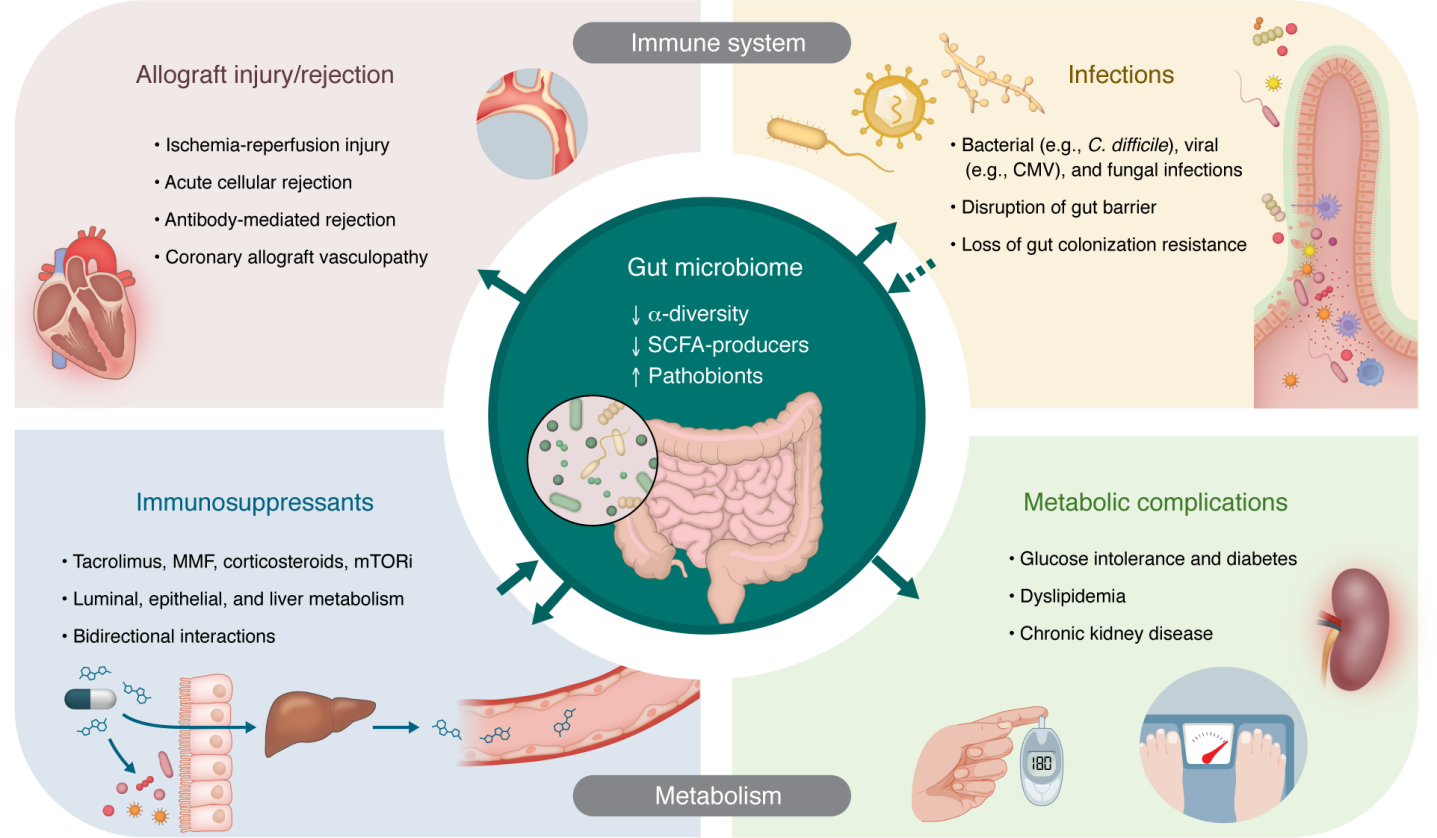
Multidimensional interactions between the gut microbiome and post-HT complications. HT-associated gut dysbiosis is characterized by reduced α-diversity, depletion of SCFA-producing bacteria, and enrichment of pathobionts. These changes bidirectionally interact with major domains affecting transplant outcomes. Allograft injury and rejection encompass ischemia/reperfusion injury, acute cellular and antibody-mediated rejection, and coronary allograft vasculopathy and are modulated by microbiome-derived metabolites and immune signaling. Infectious complications arise from gut barrier disruption and loss of colonization resistance, increasing susceptibility to bacterial, viral, and fungal pathogens. Metabolic complications including glucose intolerance, diabetes, dyslipidemia, and chronic kidney disease are influenced by alterations in microbial metabolite production and bile acid metabolism. Immunosuppressant pharmacology demonstrates bidirectional interactions, with drugs (tacrolimus, MMF, corticosteroids, mTORi) affecting microbiome composition, while microbial metabolism of immunosuppressants occurs in the luminal compartment, with microbial metabolites affecting epithelial and hepatic compartments influencing drug bioavailability and efficacy. The immune system integrates signals across all domains. Bidirectional arrows emphasize the dynamic, modifiable nature of these interactions, highlighting the gut microbiome as a therapeutic target to optimize post-transplant outcomes. MMF, mycophenolate mofetil; mTORi, mammalian target of rapamycin inhibitors; SCFA, short-chain fatty acid.

Obligatory immunosuppression used to mitigate these alloimmune complications predisposes to other morbid complications. Infections, some of the most common and serious post-HT complications, affect over half of recipients within the first post-transplant year, causing 20% of post-HT deaths (8–10). Malignancy affects over 20% of HT recipients by 10 years and is the leading cause of mortality beyond 3–5 years post-HT (11, 12). Immunosuppression also predisposes to chronic renal dysfunction and several metabolic complications (diabetes, dyslipidemia, etc.) (13, 14). With its dense connections with the host immune system and metabolism, the gut microbiome plays an important modulator role in many of these processes (Figure 1).

## Environment-microbiome-host crosstalk: a powerful modulator of host physiology

**Figure 2:**
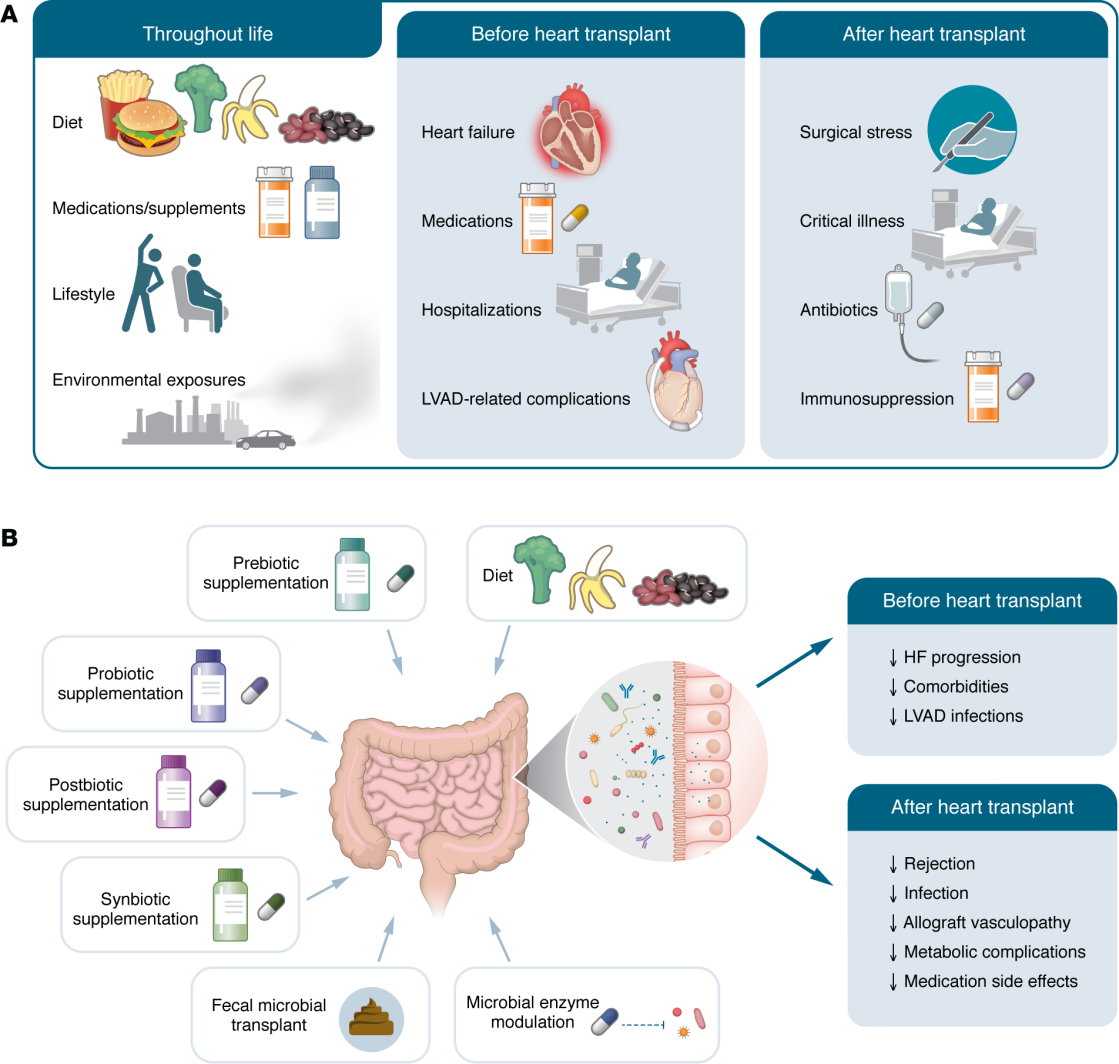
Gut microbiome modulators across the HT continuum and microbiome-targeted therapeutic strategies. (**A**) Exposures throughout life, including diet and lifestyle choices, shape the gut microbiome. Prior to HT, several factors influence the gut microbiome, including HF progression, medications, and hospitalizations, as well as left ventricular assist device–related (LVAD-related) complications. With HT, new influences are introduced, including surgical stress, critical illness, and intensive medication regimens comprising antibiotics and immunosuppressants, all of which contribute to further alterations of the gut microbiome. (**B**) The gut microbiome’s malleability presents therapeutic opportunities to restore microbial homeostasis and mitigate transplant-related complications. Interventions include diet, supplementation with prebiotics (fiber and other complex carbohydrates selectively digestible by the gut microbiota), probiotics (live microbes that when administered in adequate amounts confer a health benefit on the host), postbiotics (preparation of microbial metabolic products and/or inactivated microbial cells or cell components that confer a health benefit), synbiotics (mixtures of prebiotics and probiotics), fecal microbiota transplant, and targeted gut microbial enzyme modulation. These strategies aim to reduce complications in the pretransplant period (e.g., HF progression and LVAD-related infections) and post-HT (e.g., rejection, infection, allograft vasculopathy).

A complex and highly personalized ecosystem, the gut microbiome interfaces closely with the host to influence physiology (15, 16). Gut microbial metabolites, comprising nearly 10% of circulating metabolites in mammals (17), modulate both local and distant organs, including the heart, kidneys, and brain (18, 19). Extrinsic factors, particularly diet (20–22) and medications (including immunosuppressants) (23–26), overwhelm host genetics in shaping the microbiome composition (27) (Figure 2A), with profound physiologic effects, including in the context of HF and HT.

Diet is a principal determinant of microbiome composition. Fiber-rich diets, including Mediterranean and plant-based diets, promote antiinflammatory microbes (e.g., *Lactobacilli*, *Bifidobacteria*, *Faecalibacterium*), increasing SCFA production (28, 29). These by-products of gut microbial fermentation of dietary fiber strengthen gut barrier integrity (30, 31), reduce systemic inflammation (32, 33), enhance insulin sensitivity (34), and protect against hypertension and cardiorenal damage (35–38). Conversely, red meat and animal products abound in precursors of microbial metabolites harmful to cardiovascular health. For example, l-carnitine, phosphatidylcholine, and choline are metabolized by gut microbes into trimethylamine, which hepatic enzymes convert to trimethylamine *N*-oxide (TMAO). Nearly undetectable in vegans, vegetarians, or omnivores after red meat cessation (39), TMAO increases gut permeability, promotes inflammation and endothelial dysfunction, impairs cholesterol metabolism, and enhances platelet activation, driving atherothrombosis (39–41). These findings establish the gut microbiome as a diet-responsive metabolic interface linking environmental exposures to host physiology, with direct implications for HT outcomes.

## HF to HT: compounded insults to gut homeostasis

Chronic HF, which precedes HT, is associated with gut dysbiosis (42–50) (Figure 2B), which parallels HF severity and predicts clinical outcomes (43, 44, 48, 51). HF-associated α-diversity loss (42–44, 48, 51) reflects depletion of beneficial SCFA producers, including *Bifidobacterium*, *Faecalibacterium*, and *Lachnospira* (44, 45, 48), and overgrowth of pathobionts, including *Shigella* and *Salmonella* (47). Functionally, this results in reduced microbiome SCFA biosynthetic capacity, with loss of acetate, butyrate, and propionate, metabolites critical for regulating systemic inflammation, cardiorenal fibrosis and repair, and HF energy metabolism (35, 52, 53). Other beneficial metabolites are similarly depleted — indole-3-propionic acid, a microbial metabolite of dietary tryptophan that strengthens the gut barrier, improves insulin sensitivity, reduces inflammation, and exerts direct cardioprotective effects (54–60), is reduced in chronic HF and associated with milder HF (48, 56, 61). For a comprehensive discussion of HF-specific microbiome alterations, we direct readers to focused reviews (50, 62).

Due to limited organ availability, nearly half of US HT recipients are bridged to transplant with a durable LVAD (63), which introduces additional microbiome perturbations. These include infection-related antibiotic exposure, nonpulsatile blood flow–related gastrointestinal architectural changes, and right-sided HF. LVAD-related infections, among the most common complications (64), predispose to post-HT infections and higher mortality, likely in part through antibiotic-induced dysbiosis (65, 66).

Post-HT gut microbiome dysregulation likely reflects the composite effects of surgical stress and critical illness, and the complex immunosuppressant and antibiotic regimens, superimposed on preexisting HF-associated dysbiosis (Figure 2C). However, clinical studies characterizing the microbiome in HT recipients are limited, with a single longitudinal investigation evaluating changes from pre- to post-transplant (Table 1). In that single-center, prospective cohort of 48 recent HT recipients, α-diversity was markedly reduced compared with healthy controls (67). Multiorgan transplant recipients and patients receiving broad-spectrum antibiotics had the lowest diversity. This reflected loss of antiinflammatory Lachnospiraceae and expansion of potentially pathogenic, pro-inflammatory Enterobacteriaceae and Enterococcaceae, which correlated with reduced fecal SCFAs and secondary bile acids (67). Both of these metabolite classes are immunomodulatory (68–73), pointing to potential pathways by which the microbiome may influence post-transplant adaptive immunity. No significant pre- to post-HT microbiome shifts were observed, though the longitudinal cohort comprised just 17 recipients (67).

Microbial diversity loss post-HT was confirmed in a cross-sectional, single-center study of 86 HT recipients (median 4.2 years post-HT), revealing diversity comparable to advanced HF and inversely correlated with systemic immune activation (51). While inflammation and oxidative stress markers were lower post-transplant than in advanced HF, endotoxemia markers remained elevated compared with patients with mild HF, suggesting ongoing gut dysbiosis (51). Taxonomically, both HF and HT shared loss of Bacteroidetes, suggesting this feature persists across the disease spectrum.

Subsequent cross-sectional, single-center evaluation of 266 HT recipients, some profiled early post-transplant, revealed diversity nadir at 1 week post-transplant, gradual recovery by 3 months, and stabilization by 1 year (74). Immediately post-HT, diversity loss appeared driven by Firmicutes depletion and Proteobacteria enrichment, with temporal recovery toward HF-like composition (74). Consistent with Firmicutes recovery, enrichment of Lachnospiraceae (depleted early post-HT, ref. 67) was observed in long-term HT recipients, though high interindividual variability limited broader taxonomic interpretation. In this study, sarcopenia index, a marker of robust skeletal muscle mass, was independently associated with α-diversity and abundance of *Roseburia inulinivorans*, a prominent SCFA producer, and inversely associated with circulating C-reactive protein, IL-6, and TNF-α (74). These findings align with preclinical evidence supporting a relationship between the microbiome and skeletal musculature, termed the gut/muscle axis, partially mediated by systemic inflammation (75).

A prospective observational cohort study of over 1,300 Dutch SOT (including 67 HT) recipients demonstrated that gut dysbiosis (defined by reduced α-diversity, dissimilarity from healthy microbiome, and increased antibiotic resistance and virulence factor genes) was associated with all-cause and cause-specific mortality (76). Increased mortality risk was associated with *Ruminococcus gnavus* abundance, while decreased risk was associated with *Bifidobacterium longum* and *Bifidobacterium* *adolescentis*. A balance between 19 bacterial species predicted all-cause mortality (76), suggesting a potential risk stratification biomarker and a potentially modifiable therapeutic target in this population.

Other studies of HT recipients have focused on circulating gut microbial metabolites, which are implicated in post-transplant physiology (Supplemental Table 1; supplemental material available online with this article; https://doi.org/10.1172/JCI207999DS1). A study of TMAO and its association with inflammation, endotoxemia, and oxidative stress in HT recipients and patients with HF and LVAD found that while TMAO initially decreased post-transplant, it progressively increased to levels comparable to those in patients with symptomatic HF (77). Concurrently, inflammation markers declined post-transplant, but endotoxemia and oxidative stress markers remained elevated. After adjustment for baseline characteristics and renal function, TMAO was no longer associated with inflammation and oxidative stress. Interestingly, TMAO was not correlated with any of the metagenomic features (77), though lack of dietary data may have obscured these relationships.

TMAO, related metabolite γ-butyrobetaine (γ-BB), and their precursors trimethyllysine (TML) and l-carnitine were assessed longitudinally in 62 HT recipients in the SCandinavian HEart transplant everolimus De novo stUdy with earLy calcineurin inhibitor avoidancE (SCHEDULE), a prospective, multicenter, randomized controlled trial (RCT) comparing early everolimus- versus cyclosporine-based immunosuppression (78). These metabolites are implicated in atherothrombotic disease and HF (39, 79, 80). Baseline levels of l-carnitine, TMAO, and TML were elevated in HT recipients compared with healthy controls. Over time, TMAO and TML decreased, though they remained mildly elevated. In contrast, γ-BB and l-carnitine increased steadily post-transplant and were independently associated with future risk of acute rejection and CAV. No immunosuppressant-specific metabolite patterns emerged (78), though the cohort was likely underpowered.

Collectively, these studies demonstrated profound post-HT gut dysbiosis characterized by reduced microbial diversity, pro-inflammatory shifts, and altered metabolite profiles associated with immune dysfunction, gut barrier impairment, frailty, and increased mortality. Notably, many of these features, including loss of α-diversity and depletion of SCFA producers like Lachnospiraceae, coupled with impaired pathogen colonization resistance and expansion of pathobionts including Enterobacteriaceae and *Enterococcus*, appear shared across SOT populations. Whether organ-specific microbial signatures exist remains unclear; while some studies suggest potential distinctions (e.g., *Escherichia* enrichment in kidney transplant, KT; ref. 81), direct cross-organ taxonomic comparisons are limited by heterogeneous study design, technical variability, variable immunosuppressive and antibiotic regimens, and, particularly for HT, small cohort sizes. These confounders underscore the need for larger, standardized, multicenter studies with harmonized protocols to definitively distinguish shared versus organ-specific dysbiotic patterns and their clinical implication. Nevertheless, consistent demonstration of dysbiosis and its association with adverse outcomes across SOT populations suggest that targeting the microbiome throughout the peritransplant period may be a promising therapeutic avenue to improve long-term outcomes in transplant recipients (Figure 2D).

## Gut microbiome and allograft rejection

### Modulation of the immune system and alloimmunity.

The gut microbiome plays an integral role in shaping the immune system (82), initially by providing a diverse antigen pool to train the developing immune system and later by directing local and systemic immune responses. Gut microbes regulate intra-epithelial lymphocyte proliferation, lymphoid tissue function, and expression of critical Toll-like receptors required for systemic pathogen recognition (83, 84). Segmented filamentous bacteria in the gut also regulate production of immunoglobulin A, the predominant antibody class in humans, through direct interaction with the gut epithelium and indirectly by regulating Th17 cells (85, 86). Other microbes, such as *Coprobacillus*, stimulate antiinflammatory IL-10–producing T cells in the small intestine (87). Gut microbiome disruption activates pro-inflammatory pathways, contributing to chronic diseases characterized by systemic inflammation, including chronic HF (43).

**Figure 3:**
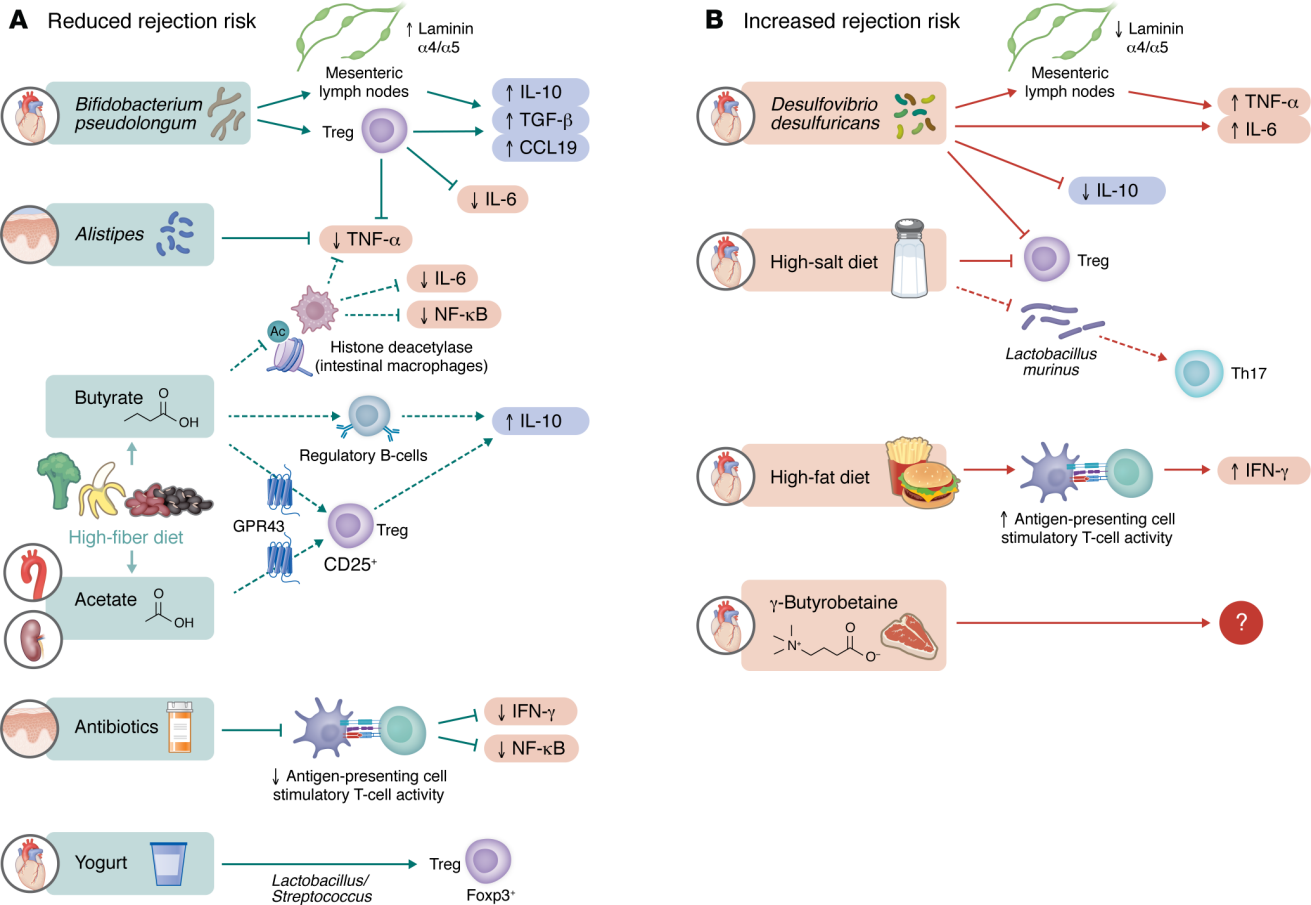
The mechanisms of gut microbiome modulation of alloimmunity and post-transplant rejection risk. (**A**) A tolerogenic environment is promoted by high-fiber diet and downstream production of SCFAs like acetate and butyrate by the gut microbiome. Acetate and butyrate promote Treg differentiation. Butyrate additionally stimulates regulatory B cells and suppresses pro-inflammatory responses by inhibiting histone deacetylase in macrophages. Beneficial bacteria, such as *Bifidobacterium* and *Alistipes*, support immune tolerance by enhancing Treg function, increasing antiinflammatory cytokines (IL-10, TGF-β), and reducing pro-inflammatory cytokines (TNF-α, IL-6). Select probiotics can further induce Tregs. (**B**) Increased rejection risk is related to heightened inflammatory tone associated with pro-inflammatory microbes such as *Desulfovibrio*, which increases inflammatory cytokines while suppressing Tregs and antiinflammatory IL-10. Unhealthy dietary habits, such as a high-salt diet, can inhibit protective bacteria, leading to the expansion of pro-inflammatory Th17 cells. High-fat diet is also associated with heightened T cell stimulation and increased rejection risk. Full lines indicate host effects demonstrated in transplant models, with colors signifying protective (green) or detrimental (red) alloimmune effects. Arrows represent stimulatory, and blunt-ended lines inhibitory, relationships. Organ icons refer to the transplanted organ (heart, kidney, skin, or aorta). Dotted lines represent immune effects demonstrated in nontransplant models, of potential relevance for alloimmunity.

The gut microbiome has also been implicated in alloimmunity, the host immune response against transplanted tissues (Figure 3). Both preclinical and clinical studies show greater post-transplant survival with “sterile” organs, such as the heart and kidney, compared with colonized organs, like the lungs and intestine (88). In mouse models, genetically identical animals with distinct gut microbiota rejected skin allografts at different rates, with genus *Alistipes* linked to slower rejection (89). Moreover, cohousing of mice with different rejection kinetics allowed for microbiome intermixing and reduced rejection rates across groups (89). Antibiotic-driven microbiome modulation pretransplant has been shown to improve allograft survival in both skin and HT mouse models, an effect mediated by attenuated type I IFN signaling in antigen-presenting cells and diminished alloreactive T cell priming (90). Additionally, the gut microbiome has been implicated in homeostatic proliferation, the process by which new memory cells emerge to fill the immunologic niche left by induction therapy. Microbiome-derived antigens partly determine which memory cells will emerge (91, 92), thus modulating overall rejection risk.

The gut microbiome modulates alloimmunity through both local and systemic facets of innate and adaptive immunity. In a fully mismatched HT mouse model, fecal microbiota transplant (FMT) from pregnant, functionally immunosuppressed mice reduced allograft inflammation and fibrosis compared with FMT from colitis-afflicted or healthy mice, improving allograft survival (93). Notably, transfer of monoculture of *Bifidobacterium pseudolongum*, enriched in the pregnant mouse microbiome, conferred a similar protective effect, suggesting that even a single species may meaningfully modulate rejection risk. These changes corresponded to increased circulating antiinflammatory IL-10 and CCL19, decreased pro-inflammatory TNF-α and IL-6, and an immunosuppressive shift in the mesenteric lymph node architecture. In contrast, transfer of *Desulfovibrio desulfuricans*, enriched in colitis-afflicted mice, promoted pro-inflammatory lymph node remodeling and a macrophage and dendritic cell inflammatory cytokine response (93).

Mechanistic studies also highlight the concept of “functional redundancy” among microbial species, whereby distinct microbial species fulfill similar physiological roles (e.g., succession between *Bifidobacterium* species) (94). Concurrently, strain-specific immune influences are evident, with divergent effects of *Bifidobacterium pseudolongum* strains in mice hypothesized to relate to their host origins, as the mouse isolate appeared to modulate Treg function, whereas the porcine-tropic strain enhanced phagocytosis and dendritic cell proliferation (95). Notably, some immune effects seem independent of long-term microbial engraftment, a phenomenon observed for both protective probiotics and pro-inflammatory *Desulfovibrio*, which promoted allograft fibrosis despite lack of persistent colonization (94). Supporting the protective role of *Bifidobacterium*, multistrain probiotic supplementation in a rat liver transplant model increased intestinal Tregs and circulating TGF-β, lowered the CD4/CD8 ratio, and ameliorated rejection-induced liver injury (96).

Gut microbial metabolites of dietary fiber, SCFAs, have also been implicated in alloimmunity. In mouse kidney and aortic transplant models, increasing SCFA levels, through either high-fiber diets or direct acetate supplementation, mitigated allograft rejection and injury while improving allograft longevity (68, 97). These effects were mediated partly through GPR43 receptor and subsequent stimulation of CD25^+^ Tregs (68). SCFAs also induce epigenetic changes; for instance, butyrate inhibits histone deacetylase in intestinal macrophages and diminishes pro-inflammatory cytokine production (98). Butyrate additionally activates regulatory, IL-10–producing B cells (99).

### Clinical implications of gut microbiome–related alloimmunity modulation.

Clinical observational studies have evaluated microbiome readouts as potential predictive biomarkers for alloimmunity. As noted above, post-transplant increases in metabolites γ-BB and l-carnitine were associated with acute rejection in HT recipients, even after adjustments for relevant clinical covariates (78). In KT recipients, risk of rejection was associated with a pretransplant Firmicutes/Bacteroidetes imbalance, reduced post-transplant α-diversity (100–102), loss of butyrate-producing taxa (e.g., *Clostridiales*, *Bifidobacterium*), and expansion of pro-inflammatory pathobionts (e.g., *Enterococcus*, *Escherichia-Shigella*) across multiple observational studies (25, 100, 101, 103). Functional dysbiosis, including enrichment of endotoxin biosynthetic pathways and reduced SCFAs, was also associated with higher rejection risk (101). Elevated circulating TMAO was also linked with kidney allograft failure and mortality, though a direct immunological mechanism remains unconfirmed (104, 105). Crucially, longitudinal studies in KT recipients demonstrate that microbiome alterations precede clinical rejection, normalizing following treatment (100). Moreover, in a prospective study of 97 KT recipients, microbiome signatures enhanced rejection prediction beyond clinical parameters alone (101).

The gut microbiome is also positioned to be a therapeutic target for mitigating rejection. Preclinical studies of kidney and aortic transplantation show reduced rejection and prolonged allograft survival with high-fiber diet (68, 97), offering a proof of concept for potential allograft protection through plant-based, fiber-rich diets (e.g., Mediterranean) with ample SCFA precursors. Fiber (prebiotic) supplementation may further enhance SCFA delivery. While these dietary approaches remain investigational in SOT recipients with no HT-specific data, an ongoing RCT in KT recipients is evaluating the feasibility and efficacy of early post-transplant prebiotic inulin fiber supplementation on systemic inflammation, immune function, and allograft outcomes (106).

Reducing red meat consumption, rich in precursors for TMAO, γ-BB, and other proatherogenic and pro-inflammatory microbial metabolites (39, 107), may be another protective intervention. Notably, while TMAO levels are elevated post-HT (77, 78), a clear association with systemic inflammation or rejection has not yet been established (78). γ-BB has been associated with increased risk of rejection and CAV post-HT (78), though it remains to be determined whether dietary red meat and animal product restriction would lower circulating γ-BB and mitigate rejection.

Other prominent features of a Western diet, such as high salt and saturated fat intake, also modulate alloimmunity. In a mouse HT model, a high-salt diet suppressed Treg proliferation and accelerated rejection via serum- and glucocorticoid-regulated kinase-1 (108). Mechanistically, a high-salt diet depleted gut *Lactobacillus murinus*, leading to Th17 cell proliferation and hypertension, effects abrogated by *L*. *murinus* probiotic supplementation both in mice and in a pilot study of healthy adults (109). Although dietary sodium restriction is routinely recommended post-HT, this recommendation is extrapolated from non-HT populations and hypertension, HF, and chronic kidney disease management guidelines. In KT at least one RCT has demonstrated marked blood pressure reduction with strict sodium control (<2.3 g/d) (110, 111). Similarly, high-fat diet in a mouse HT model enhanced antigen-presenting cell activity and increased IFN-γ production, accelerating rejection (112).

Conversely, fermented foods represent a promising avenue for beneficial host immunomodulation. Foods like yogurt, kefir, kombucha, sourdough, sauerkraut, tempeh, and others, produced through controlled and desirable microbial growth (113), represent a source of beneficial microbial metabolites (including SCFAs) and, in some cases, live microbes. In healthy adults, a fermented food–rich diet increased microbiome α-diversity and decreased circulating inflammatory markers (114). In a mouse HT model, treatment with a probiotic yogurt containing live lactic acid bacteria increased the number of CD4^+^Foxp3^+^ Tregs, extending heart allograft survival (115). While the risk of iatrogenic infection in immunosuppressed recipients requires careful consideration, fermented foods may offer net benefits, particularly as immunosuppression is tapered.

## Gut microbiome and implications for post-transplant infections

Infections represent a major post-transplant complication (8–10). The gut microbiome modulates vulnerability to infection through systemic immunomodulation and by serving as a pathobiont reservoir. Central to this relationship is colonization resistance, the microbiome’s collective ability to prevent pathogen establishment, which becomes profoundly impaired post-transplant (116). Loss of SCFA producers disrupts the metabolic barrier maintaining an acidic, pathogen-hostile environment while impairing epithelial integrity and antimicrobial peptide production (117–119). Depleted secondary bile acid producers eliminate key metabolites that inhibit pathogen growth (120, 121). Additionally, immunosuppression disrupts secretory IgA-mediated governance in pediatric SOT recipients (122). This multifactorial collapse of colonization resistance leads to enrichment of antibiotic resistance genes and virulence factors that persist years post-transplant (123).

Clinically, microbiome disruption manifests as enrichment of potential pathogens, including multidrug-resistant bacteria (MDRB), which has been associated with infection risk in observational studies. In a single-center prospective cohort of 121 HT recipients, pretransplant *Enterococcus* and *Enterobacterales* enrichment was associated with early post-transplant infections, while an infection-free course was associated with preserved Bacteroidetes, Lachnospiraceae, and Ruminococcaceae (124). Low pretransplant α-diversity similarly correlated with infection risk. In another small observational study of 20 HT recipients, recipients with post-transplant infections exhibited lower α-diversity and *Enterococcus faecium* overgrowth, compared with noninfected recipients, who had enrichment of butyrate producers Lachnospiraceae, including *Blautia*, *Dorea*, and *Lachnospira* (125). However, whether these patterns are causes or consequences of infections and associated antibiotic exposure remains unclear.

Similar associations have been observed across transplant types: higher abundances of *Anaerotruncus*, *Faecalibacterium*, and other SCFA producers were associated with fewer urinary, respiratory, and viral infections in KT and allogeneic stem cell transplant recipients (126–129). Conversely, *Enterococcus* and *Escherichia* enrichment in KT recipients was associated with bacteriuria and urinary tract infections with phylogenetically matched organisms (25, 126), suggesting the microbiome serves as a pathogen reservoir. Supporting the temporal relationship between microbiome alterations and infection risk, low α-diversity and *Enterococcus* enrichment pretransplant predicted MDRB colonization within the first year after liver transplant (130), which was previously linked to clinical infections in this population (131).

These findings support microbiome modulation as a potential infection prevention strategy. Meta-analysis of multiple RCTs in liver transplant recipients demonstrated that prophylactic probiotics reduced infection rates from 33% to 7% and shortened hospital stays, albeit without affecting mortality (132). Combined *Lactococcus*, *Lactobacillus*, and *Bifidobacterium* probiotic supplementation similarly reduced infection risk in yet another double-blind RCT of liver transplant recipients (133). Beyond probiotics, FMT has shown promise for targeted pathogen control: a small RCT in KT recipients demonstrated efficacy for MDRB decolonization through mechanisms involving competitive strain exclusion (134). Whether such microbiome-directed interventions may benefit HT recipients warrants dedicated clinical trials.

### Clostridioides difficile infection.

A quintessential example of failed colonization resistance, *Clostridioides* *difficile* infection (CDI) remains another important post-transplant complication. HT recipients have the highest incidence of CDI among SOT recipients, with an associated 7.7-fold mortality increase (135–137). While gut microbiome signatures can predict CDI in nontransplant populations (138, 139), similar approaches have not been evaluated in transplant recipients. CDI preventative strategies in transplant patients remain controversial (140, 141). Although prophylactic *Lactobacillus plantarum* V299 probiotic reduced risk of CDI and post-transplant diarrhea in KT recipients (141), this approach carries iatrogenic infection risk, with reported cases of *Lactobacillus* bacteremia and asymptomatic colonization in thoracic transplant recipients receiving routine probiotic prophylaxis (142, 143).

No RCTs have evaluated CDI therapeutics specifically in SOT recipients. Guidelines recommend treatment with oral vancomycin or fidaxomicin, with FMT considered for recurrent CDI (144). Retrospective data suggest efficacy similar to that in immunocompetent hosts (145), yet pediatric HT case reports reveal a high-risk profile (146–148). While one case reported durable CDI resolution following FMT (147), another described a poor outcome where post-FMT a patient developed DSAs, severe mixed rejection, and CAV requiring retransplantation, complicated by CDI recurrence (148). These divergent outcomes underscore the urgent need for prospective studies to establish safety and efficacy of microbiome-based CDI interventions in HT recipients.

### CMV infection.

CMV is the most common viral infectious culprit following HT, with substantial morbidity (9). CMV increases SOT rejection risk (149–151), likely through both reduced immunosuppression during active infection and CMV’s multifaceted immunomodulatory effects, including endothelial injury, which promotes allorecognition and non-HLA antiendothelial antibodies, CMV donor molecular mimicry, and altered macrophage function (152–155). Reflecting its endothelial tropism, CMV also portends higher risk of CAV in HT recipients (156) and opportunistic infections (157, 158).

The bidirectional CMV-microbiome relationship post-transplant remains poorly defined. Preclinical evidence demonstrates that CMV infection induces gut dysbiosis, transiently decreasing Firmicutes and increasing Bacteroidetes (159). In rhesus macaques, CMV reduces SCFA-producing *Butyrivibrio*, *Sarcina*, and *Blautia* while enriching pathogenic *Streptococcus*. Mechanistically, CMV-induced gut dysbiosis may involve IL-15 overexpression and Th17/Treg imbalance, alongside local gut inflammation with epithelial damage and elevated TNF-α, IFN-γ, and IL-6, which may potentiate alloimmune responses (160).

Clinical data are limited. In KT recipients, higher abundance of butyrate-producing microbes was associated with a trend toward lower risk of CMV viremia in the first post-transplant year (161). While preliminary, these findings suggest that the gut microbiome may represent a modifiable factor influencing CMV-related complications in SOT.

### Impact of the gut microbiome on CAV.

In HT recipients, CAV is a principal driver of long-term allograft failure (162, 163). Characterized by diffuse, concentric narrowing of large epicardial and smaller intra-myocardial arteries, CAV pathophysiology involves endothelium activation, smooth muscle proliferation, and extracellular matrix deposition driven by alloimmune and cardiometabolic factors (7, 164–166). This positions the gut microbiome, which influences both alloimmunity and metabolism, as a plausible CAV modulator. However, direct evidence linking the microbiome to CAV remains sparse. The single longitudinal microbiome study in HT reported that increases in gut microbial metabolites γ-BB and TML between baseline and 3 years post-transplant were associated with increased total atheroma volume (78). There were no correlations with other CAV metrics or differences between everolimus- and cyclosporine-treated cohorts in this likely underpowered comparison. Elucidating the mechanistic basis and clinical relevance of this association will require larger longitudinal cohorts with granular clinical phenotyping and integrated multiomic profiling to dissect the microbiome, immune, and metabolic interactions in CAV progression.

## The gut microbiome and immunosuppressants

The relationship between the microbiome and medications is bidirectional and clinically meaningful in HT recipients. The microbiome modulates immunosuppressant pharmacokinetics, altering drug efficacy and toxicity, while immunosuppressants shift microbiome composition, contributing to their immunomodulatory effects. Elucidating this interplay is essential for personalizing immunosuppressive regimens and improving HT outcomes.

### Tacrolimus.

**Figure 4:**
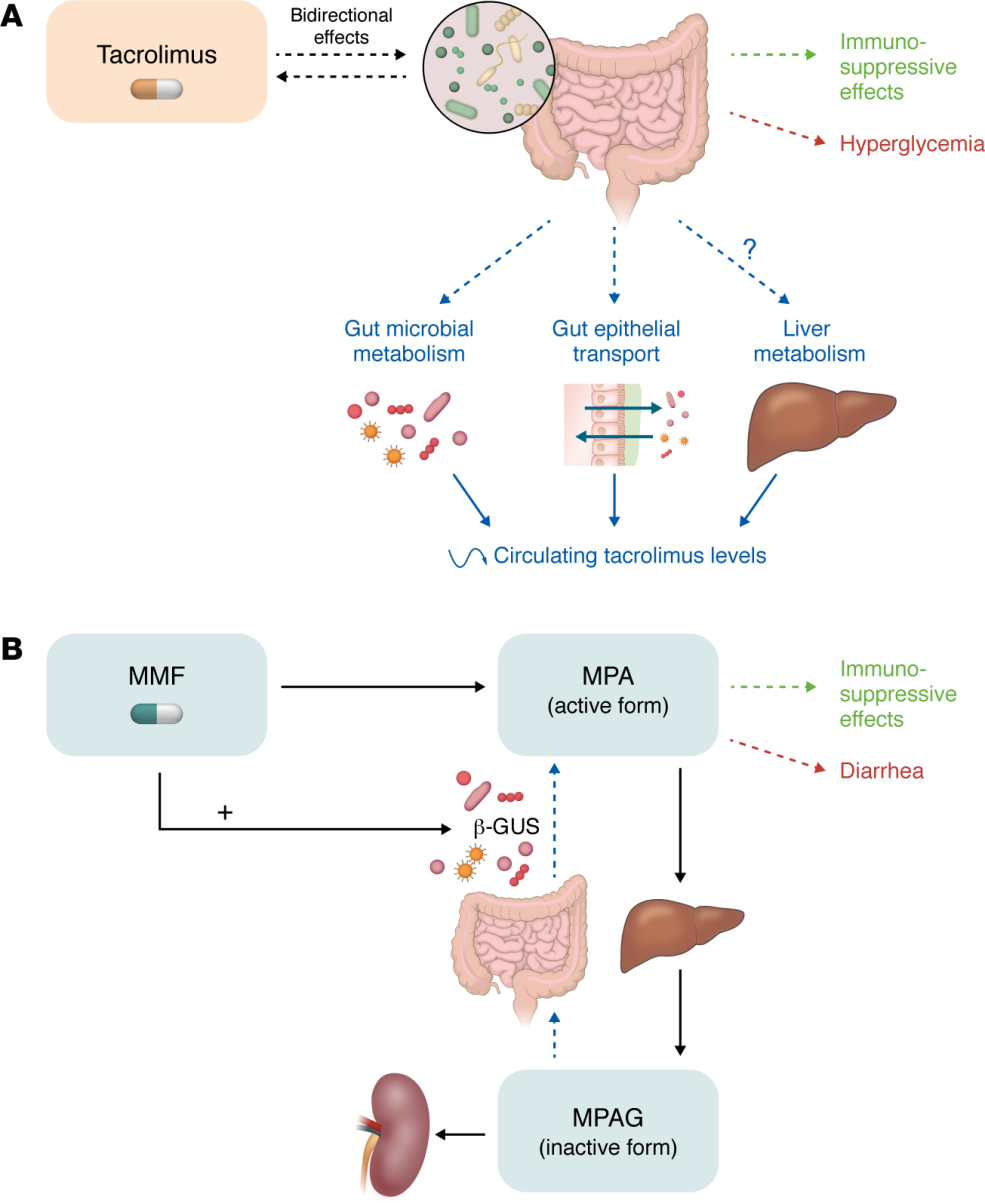
Bidirectional interactions between the gut microbiome and commonly used immunosuppressants after HT. (**A**) Gut microbes metabolize tacrolimus into its less potent metabolite, in addition to affecting transport of tacrolimus among the gut lumen, epithelium, and circulation. The gut microbiome affects expression of liver-metabolizing enzymes, which may also affect systemic tacrolimus levels. (**B**) MMF is converted by the host to its active form, mycophenolic acid (MPA). MPA is modified by the liver into inactive MPA-glucuronide (MPAG), which is either excreted by the kidneys or delivered back into the gut lumen, where gut microbes, via the β-glucuronidase (β-GUS) enzyme, metabolize it back to active MPA. MPA mediates MMF-related immunosuppressive effects as well as intestinal inflammation and diarrhea, common MMF side effects that are entirely absent in germ-free animals. MMF also alters gut microbiome composition by stimulating growth of microbes that produce β-GUS. Blue lines denote effects on drug metabolism. Green lines signify immunosuppression-related effects. Red lines refer to adverse effects.

The calcineurin inhibitor (CNI) tacrolimus is the foundational immunosuppressant for HT recipients. Its narrow therapeutic window and high pharmacokinetic variability pose clinical challenges, risking underimmunosuppression and rejection, or overexposure and toxicity. The gut microbiome contributes to this variability through direct drug metabolism (Figure 4A). Common gut microbes, including *Faecalibacterium prausnitzii*, metabolize tacrolimus to less potent metabolites that crossreact with standard immunoassays, confounding therapeutic monitoring (167). This phenomenon can lead to overestimation of biologically active drug concentrations, potentially masking subtherapeutic immunosuppression. Clinically, higher abundances of *Faecalibacterium prausnitzii* and related *Subdoligranulum* have been associated with increased tacrolimus dose requirements in KT and HT recipients, respectively (168, 169).

The microbiome also modulates host metabolism, affecting tacrolimus disposition. Antibiotic-induced microbiome shifts increase mouse gut epithelium ABCB1 transporter expression, suppressing tacrolimus recirculation and reducing systemic tacrolimus levels (170). Gut microbial metabolites, particularly SCFAs, function as epigenetic regulators altering hepatic cytochrome P450 expression, influencing drug clearance. Additionally, microbiome-produced indoxyl sulfate modulated host *CYP3A4* expression with interindividual variability and context dependence (171).

The microbiome-tacrolimus interaction is bidirectional. Tacrolimus increases gut permeability, potentially facilitating microbial product translocation that influences systemic immunity (172–174). In mice, tacrolimus enriched *Allobaculum*, *Bacteroides*, and *Lactobacillus*, shifting SCFA production (26). Critically, these changes mechanistically contribute to tacrolimus pharmacodynamic effects. For example, combination of low-dose tacrolimus and FMT from high-dose tacrolimus-treated mice fully recapitulated the high-dose tacrolimus treatment effects by expanding Treg populations, reducing pro-inflammatory cytokines, and prolonging skin allograft survival (26). These landmark findings established a paradigm wherein the gut microbiome is not a passive bystander but an active intermediary in the drug’s mechanism of action.

The microbiome also mediates tacrolimus adverse effects like post-transplant diabetes mellitus (PTDM) (175). Specific gut microbiome signatures are associated with PTDM risk in liver transplant and KT recipients (176, 177). Preclinical studies have demonstrated that tacrolimus upregulates bacterial β-glucuronidase (GUS) activity, alters bile acid metabolism, activates ileal farnesoid X receptor, and suppresses glucagon-like peptide-1 secretion, impairing glucose homeostasis (178). Importantly, inhibiting bacterial GUS with vancomycin or by supplementing the SCFA butyrate ameliorated tacrolimus-induced hyperglycemia in preclinical models (179, 180).

These studies highlight a complex triad involving tacrolimus, gut microbiome, and host immunity and metabolism. Defining these interactions in human cohorts is critical for optimizing the efficacy and safety of this essential immunosuppressant.

### MMF.

MMF, another key post-HT immunosuppressant, demonstrates bidirectional microbiome interactions that profoundly affect its efficacy and toxicity (Figure 4B). Host tissues metabolize MMF to active mycophenolic acid (MPA), which undergoes hepatic glucuronidation to inactive MPA-glucuronide (MPAG). MPAG is renally excreted or undergoes enterohepatic recirculation, where gut microbial β-glucuronidases reactivate it to MPA, accounting for 30%–40% of circulating MPA (181). Critically, MMF selectively expands GUS-expressing bacteria, creating a feed-forward loop that increases MPA levels (182).

These interactions have profound clinical consequences. In mice, colonic inflammation, diarrhea, and weight loss occur only in MMF-treated conventional but not germ-free animals, establishing that gastrointestinal toxicity, one of the most common MMF side effects, is entirely microbiome dependent (183). Multiple mechanisms contribute. First, MMF-mediated expansion of GUS-expressing bacteria elevates colonic MPA levels; in mice, vancomycin selectively eradicates these bacteria and prevents gastrointestinal toxicity (182). Second, MMF reduces α-diversity while enriching pathogenic *Escherichia* and *Shigella*, enhancing microbial endotoxin biosynthesis and increasing its fecal and circulating levels, driving severe colonic inflammation (183). Third, MMF reduces gut and systemic SCFA levels (184), with potential consequences for alloimmunity and metabolic homeostasis.

Clinical studies corroborate these preclinical findings. Among 139 KT recipients, MMF was the dominant driver of microbiome dysbiosis, causing α-diversity loss, depletion of antiinflammatory Actinobacteria and SCFA producers, and expansion of pro-inflammatory Proteobacteria (185). In 97 KT recipients, elevated fecal GUS activity was strongly associated with prolonged post-transplant diarrhea (186). Clinical microbiome-targeted interventions remain unexplored, though proof of concept exists in octyl gallate, a food additive with antioxidant and GUS-inhibitory properties, which prevents MMF gastrointestinal toxicity in mice (187). Whether MMF-mediated microbiome shifts contribute to other MMF adverse effects, including myelosuppression (188), warrants investigation.

### Corticosteroids.

Corticosteroids, another integral post-HT immunosuppressant drug class, demonstrate reciprocal microbiome interactions through active microbial metabolism and microbiome alterations. Gut *Clostridium steroidoreducens* expresses steroid reductases that inactivate exogenous steroids and reduce their bioavailability (189). One clinical study associated steroid treatment with gut enrichment of *Streptococcus salivarius*, typically an oral commensal, potentially signaling gut dysbiosis (190). However, human data on steroid-induced microbiome changes remain limited.

Mouse models provide mechanistic insight. Steroid treatment increased *Anaerobacterium* while depleting *Eisenbergiella*, *Alistipes*, and *Clostridium XIVb*, predicted to diminish SCFA production and compromise immune regulation (191). Comparative analysis in mice revealed the most pronounced microbiome shifts with prednisolone, characterized by reduced Bacteroidetes and expanded Firmicutes (192). This was accompanied by decreased gut antimicrobial defensins Reg3γ and IL-22, *Escherichia coli* overgrowth, and colonization with its uropathogenic strain. Notably, these microbiome effects appeared dependent on baseline microbiome profiles (192).

Intriguingly, some corticosteroid immunosuppressive effects may be microbiome mediated. In a lupus mouse model, prednisone’s systemic immunomodulatory effects were comparable to those elicited by FMT from prednisone-treated mice (193). These findings highlight critical knowledge gaps and raise questions about whether baseline microbiome profiles may influence steroid efficacy and toxicity.

### mTOR inhibitors.

Sirolimus (rapamycin) and everolimus, mTOR inhibitors, are frequently used post-HT to reduce malignancy risk and feature prominently in CNI-sparing regimens to mitigate renal dysfunction.

Multiple preclinical studies demonstrate that mTOR inhibitors profoundly alter gut microbiome composition with functional immunologic consequences. Sirolimus induces dysbiosis characterized by increased pro-inflammatory Proteobacteria and decreased antiinflammatory *Akkermansia*, with associated shifts in microbiome lipid metabolism and immune pathways (194). Sirolimus also compromises gut barrier integrity, increasing gut permeability and circulating pro-inflammatory cytokines (194). Time-dependent sirolimus-induced microbiome shifts include reduced α-diversity and increased Bacteroides/Firmicutes ratio with elevated gut luminal amino acid availability, paralleling early pro-inflammatory to protolerogenic transitions through modulated lymph node architecture and Treg distribution (195). mTOR inhibitor–induced shifts in Firmicutes, Acidobacteria, and Bacteroidetes correlated with Peyer’s patch and splenic T cell populations, some associated with prolonged lifespan (196).

The microbiome also mediates the metabolic complications associated with mTOR inhibitors. In mice fed high-fat diet, sirolimus induced microbiome alterations, worsening gut inflammation and glucose intolerance, which were mitigated by natural polyphenol antioxidant resveratrol (197). Similarly, probiotic *Lactobacillus rhamnosus* HN001 alleviated sirolimus-induced systemic inflammation, dyslipidemia, and insulin resistance (194), suggesting potential microbiome-targeted therapeutic interventions for managing mTOR inhibitor–associated metabolic toxicity in HT recipients.

### Implications of gut microbiome–immunosuppressant interactions for HT.

Immunosuppressants are indispensable after HT, yet emerging evidence reveals that they function within a tripartite drug-microbiome-host system. The microbiome actively determines immunosuppressant pharmacokinetics, pharmacodynamics, and toxicity.

These insights suggest translational opportunities for precision immunosuppression management to optimize allograft protection while minimizing adverse effects. However, current evidence is derived predominantly from preclinical models and non-HT populations, necessitating dedicated studies in HT recipients to validate these associations and establish clinical utility. Microbiome profiling, including quantifying gut *Faecalibacterium prausnitzii* (167–169) or GUS activity (186), could facilitate therapeutic drug monitoring and mitigate adverse effects. Targeted microbiome modulation offers a strategy to decouple efficacy from toxicity. Preclinical proof of concept includes the findings that butyrate supplementation prevents tacrolimus-associated hyperglycemia (180), GUS inhibition mitigates MMF-related diarrhea (182, 187), and probiotics ameliorate sirolimus-related metabolic complications (194). As noted above, FMT from tacrolimus-treated mice recapitulates immunosuppressive effects at lower drug exposure (26), suggesting microbiome-targeted therapies might enhance allograft tolerance while reducing drug burden and associated infectious and malignant complications.

## Current challenges and a roadmap to clinical translation

Despite growing interest in the gut microbiome as a contributor to post-HT outcomes, clinical translation remains limited by practical and evidentiary gaps. Most studies are small and cross-sectional, lacking the longitudinal sampling needed to distinguish baseline variation from peritransplant perturbations. Time-varying confounders including antibiotics, immunosuppression, infections, hospitalization, diet, and comorbidities obscure causal relationships and limit reproducibility.

Many proposed microbiome biomarkers remain difficult to generalize, relying on taxonomic signals that vary across populations and sequencing platforms. Clinically useful biomarkers will likely require individualized, function-based approaches (microbial pathways, metabolites, and host-microbe interactions) rather than taxonomy alone. Moreover, transplant-specific data on microbiome-directed interventions are sparse. A systematic review identified only 5 RCTs of prebiotics/probiotics in liver and KT recipients, with substantial bias (198). Early-phase trials for FMT for SOT-related complications are emerging (NCT06708676), but HT-specific trials remain absent. Safety concerns are paramount given live biotherapeutic-related iatrogenic infection risk in immunosuppressed patients.

Moving microbiome science toward HT clinical practice requires stepwise evidence-building prioritizing standardization, longitudinal data, and clinically meaningful endpoints. Multicenter cohort infrastructure with harmonized serial biospecimen collection is essential, linked to curated clinical datasets capturing immunosuppressive regimens, antibiotic exposures, rejection and infection episodes, hospitalizations, comorbidities, and nutrition. Existing biobanks, consortia, and clinical trials should be strategically leveraged to incorporate microbiome-relevant sampling. Initial examples of such integration exist, including TransplantLines Biobank and Cohort Study (NCT03272841; focused on SOT in general; ref. 199) and BIOMARGIN (NCT02832661; focused on KT recipients; ref. 200), though comprehensive, multicenter microbiome biobanking at scale in transplantation remains an unmet need.

Analytics must advance beyond descriptive profiling toward functional, actionable readouts. Integrative, multiomics frameworks linking microbiome variation to host immunity and metabolism can identify pathways relevant to rejection, infection, and metabolic complications. Critically, candidate biomarkers must map to clear clinical decisions and undergo cross-site validation.

With this foundation, adequately powered RCTs can test well-characterized microbiome interventions (Figure 2D), using standardized endpoints, applying safety monitoring, and accounting for relevant effect modifiers. Reverse-translational work using in vitro and animal models should test causality and refine targets iteratively.

Beyond recipient-focused interventions, frontier approaches may target earlier transplant stages. Machine perfusion technology enables ex vivo therapeutic intervention. Supplementing perfusion solutions with gut-derived microbial metabolites like SCFAs and indole-3-propionic acid, which protect against IRI in preclinical nontransplant models and serve as myocardial fuel substrates (201–206), represents a novel strategy to reduce early graft damage. The donor gut microbiome may also influence allograft quality through circulating microbial metabolites that may prime donor myocardium for post-HT inflammatory response. Whether profiling donor microbial metabolites or optimizing donor gut health through targeted interventions can improve HT outcomes warrants further investigation.

Finally, progress will depend on collaboration and patient engagement. A dedicated transplant microbiome consortium could enable large studies with harmonized methods, accelerating validation. Patient-facing tools tracking diet, symptoms, and medications may strengthen adherence in intervention studies and provide context for interpreting microbiome dynamics. Together, these efforts can shift the field from associations to validated, safe microbiome-informed strategies for HT recipients, enabling precision and personalized transplant care to improve survival and quality of life for HT recipients.

## Conflict of interest

The authors have declared that no conflict of interest exists.

## Funding support

This work is the result of NIH funding, in whole or in part, and is subject to the NIH Public Access Policy. Through acceptance of this federal funding, the NIH has been given a right to make the work publicly available in PubMed Central.

- NIH K08 HL164881 (PM).

## Supplementary Material

Supplemental data

## Figures and Tables

**Table 1:**
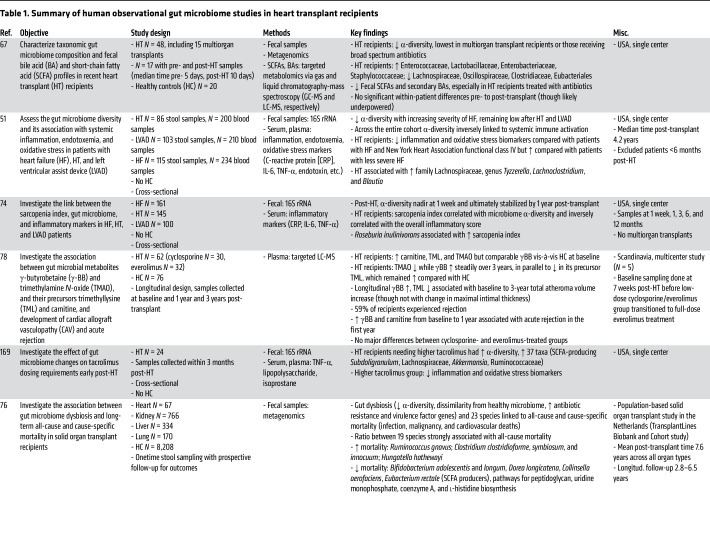
Summary of human observational gut microbiome studies in heart transplant recipients

## Version 1 08/17/2026

Electronic publication
