# Supplementary material for: The gut microbiome and allograft outcomes: implications for heart transplantation
Source: J Clin Invest. 2026 Aug 17;136(16):e207999. doi: 10.1172/JCI207999 (PMC13476144; doi:10.1172/JCI207999)
Supplement: Supplementary file 1 [file jci-136-207999-s048.pdf]

Online supplementary material regarding the review manuscript

The gut microbiome and allograft outcomes: implications for heart transplantation

Ivan Đuran[1,2], W. H. Wilson Tang[3,4], Petra Mamic[5#]

[1]University of Zagreb School of Medicine, Zagreb, Croatia

[2]Public Health Center (Dom Zdravlja) Zagreb-Centar, Zagreb, Croatia

[3]Kaufman Center for Heart Failure Treatment and Recovery, Heart, Vascular and Thoracic Institute, Cleveland Clinic, Cleveland, OH 44195

[4]Department of Cardiovascular and Metabolic Sciences, Lerner Research Institute, Cleveland Clinic, Cleveland, OH 44195

[5]Division of Cardiovascular Medicine, Department of Medicine, Stanford University School of Medicine, Stanford, CA 94305, USA

[#]: To whom correspondence should be addressed: mamic@stanford.edu, pmamic1@gmail.com

Supplemental Table 1. Relevant gut microbiome metabolites in transplantation.

Gut microbial metabolite			Transplant-relevant studies
Short chain fatty acids (dietary fiber metabolites)		**Acetate**	Human studies:<br>• ↓ Fecal levels in HT recipients (1)<br>Preclinical models:<br>• ↑ Regulatory T cell (Treg) and donor-specific tolerance via GPR43 activation (2)<br>• ↓ Rejection risk in aortic transplant (mouse) (2)<br>• ↓ Rejection risk in kidney transplant (KT) (via GPR43) (mouse) (3)<br>• ↓ Production with tacrolimus (mouse) (4)
		**Butyrate**	Human studies:<br>• ↓ Fecal levels in HT recipients (1)<br>• Inverse association between butyrate-producing bacteria and respiratory viral infections in KT and stem-cell transplant (5, 6)<br>Preclinical models:<br>• ↓ Tacrolimus-induced hyperglycemia (mouse) (7)
		**Propionate**	Human studies:<br>• ↓ Fecal levels in HT recipients (1)
Dietary L-carnitine, phosphatidylcholine, and choline metabolites		**Trimethylamine-N-oxide (TMAO)**	Human studies:<br>• Early post-HT ↑, followed by partial ↓, but persistent ↑ compared to healthy controls (HC) (8)<br>• ↓ Early post-HT (1-week and 1-month) compared to HF, later increasing to levels comparable to symptomatic HF (>6 month) (9)<br>• ↑ Risk of graft failure in KT (10)<br>• ↑ In liver transplant recipients, and associated with higher mortality (11)<br>Preclinical models:<br>• Accelerates graft-versus-host-disease (GVHD) progression (via M1 macrophage polarization) (12)
		**γ-Butyrobetaine (γ-BB)**	Human studies:<br>• In HT recipients, ↑ steadily over 3 years of follow-up, associated with progression in atheroma volume and risk of acute rejection (8)
Dietary amino acid metabolites	Tryptophan	**Indole-3-propionic acid (IPA)**	Human studies:<br>• ↑ Associated with improved graft function early post-KT (13)<br>• ↓ In patients with GVHD after allogeneic stem cell transplantation (alloSCT), along with other microbial tryptophan metabolites (14)
		**Indoxyl sulfate (IS)**	Human studies:<br>• Low urinary levels of IS early after alloSCT associated with worse survival (15)<br>• ↓ at onset of GVHD (14)
	Phenyl-alanine	**Phenylacetyl-glutamine (PAGln)**	Human studies:<br>• ↑ Arterial stiffness in KT recipients, biomarker of vascular risk (16)
Bile acids			Human studies:<br>• ↓ Fecal levels of secondary BAs lithocholic acid (LCA), deoxycholic acid, and isodeoxycholic acid (isoDCA), and modified LCAs (alloisoLCA and 3-oxoLCA) in HT recipients (1)<br>• LCA inversely associated with duration of pre-HT hospitalization (1)<br>Preclinical models:<br>• ↓ BA pool in acute GVHD (17)<br>• Tauroursodeoxycholic acid ↓ activity of antigen-presenting cells (APCs) and ↓ GVHD severity in multiple mouse models (17)<br>• Secondary BA isoDCA ↓ APC activity, ↑ Foxp3 induction, ↑ colonic retinoid-related orphan receptor-γt (RORγt)+ Tregs, via FXR-dependent mechanism (18)<br>• 3-OxoLCA ↓ Th17 cell differentiation via RORγt-dependent mechanism (19, 20)<br>• IsoalloLCA ↑ Treg differentiation via mitochondrial reactive oxygen species and ↑ Foxp3 expression (19-21)

Abbreviations: 3-oxoLCA, 3-oxolithocholic acid; alloSCT, allogeneic stem cell transplantation; alloisoLCA, alloisolithocholic acid; BA, bile acid; FXR, farnesoid X receptor; GPR43, G-protein coupled receptor 43; GVHD, graft-versus-host-disease; HC, healthy controls; HF, heart failure;

HT, heart transplant; IPA, indole-3-propionic acid; IS, indoxyl sulfate; isoDCA, isodeoxycholic acid; KT, kidney transplant; LCA, lithocholic acid; PAGln, phenylacetylglutamine; RORγt, retinoid-related orphan receptor-γt; TMAO, trimethylamine-N-oxide;; γ-BB, γ-butyrobetaine

Supplemental references

1. Dela Cruz M, et al. Reduced immunomodulatory metabolite concentrations in peri-transplant fecal samples from heart allograft recipients. *Front Transplant*. 2023;2. https://doi.org/10.3389/frtra.2023.1182534.
2. Rey KM, et al. Dysbiosis of the Female Murine Gut Microbiome Exacerbates Neutrophil-mediated Vascular Allograft Injury by Affecting Immunoregulation by Acetate. *Transplantation*. 2022;106(11):2155.
3. Wu H, et al. Gut Microbial Metabolites Induce Donor-Specific Tolerance of Kidney Allografts through Induction of T Regulatory Cells by Short-Chain Fatty Acids. *J Am Soc Nephrol*. 2020;31(7):1445–1461.
4. Zhang Z, et al. Immunosuppressive effect of the gut microbiome altered by high-dose tacrolimus in mice. *Am J Transplant*. 2018;18(7):1646–1656.
5. Haak BW, et al. Impact of gut colonization with butyrate-producing microbiota on respiratory viral infection following allo-HCT. *Blood*. 2018;131(26):2978–2986.
6. Lee JR, et al. Butyrate-producing gut bacteria and viral infections in kidney transplant recipients: A pilot study. *Transpl Infect Dis*. 2019;21(6):e13180.
7. Jiao W, et al. Butyric acid normalizes hyperglycemia caused by the tacrolimus-induced gut microbiota. *Am J Transplant*. 2020;20(9):2413–2424.
8. Trøseid M, et al. The carnitine-butyrobetaine-TMAO pathway after cardiac transplant: Impact on cardiac allograft vasculopathy and acute rejection. *J Heart Lung Transplant*. 2019;38(10):1097–1103.
9. Yuzefpolskaya M, et al. Levels of Trimethylamine N-Oxide Remain Elevated Long Term After Left Ventricular Assist Device and Heart Transplantation and Are Independent From Measures of Inflammation and Gut Dysbiosis. *Circ Heart Fail*. 2021;14(6):e007909.
10. Flores-Guerrero JL, et al. Association of Circulating Trimethylamine N-Oxide and Its Dietary Determinants with the Risk of Kidney Graft Failure: Results of the TransplantLines Cohort Study. *Nutrients*. 2021;13(1):262.
11. Trillos-Almanza MC, et al. Circulating Trimethylamine-N-Oxide Is Elevated in Liver Transplant Recipients. *International Journal of Molecular Sciences*. 2024;25(11):6031.

12. Wu K, et al. The gut microbial metabolite trimethylamine *N*-oxide aggravates GVHD by inducing M1 macrophage polarization in mice. *Blood*. 2020;136(4):501–515.
13. Viejo-Boyano I, et al. Early Metabolomic Profiling as a Predictor of Renal Function Six Months After Kidney Transplantation. *Biomedicines*. 2024;12(11):2424.
14. Michonneau D, et al. Metabolomics analysis of human acute graft-versus-host disease reveals changes in host and microbiota-derived metabolites. *Nat Commun*. 2019;10:5695.
15. Weber D, et al. Low urinary indoxyl sulfate levels early after transplantation reflect a disrupted microbiome and are associated with poor outcome. *Blood*. 2015;126(14):1723–1728.
16. Yang H-H, et al. Serum Phenylacetylglutamine among Potential Risk Factors for Arterial Stiffness Measuring by Carotid-Femoral Pulse Wave Velocity in Patients with Kidney Transplantation. *Toxins (Basel)*. 2024;16(2):111.
17. Haring E, et al. Bile acids regulate intestinal antigen presentation and reduce graft-versus-host disease without impairing the graft-versus-leukemia effect. *Haematologica*. 2021;106(8):2131–2146.
18. Campbell C, et al. Bacterial metabolism of bile acids promotes peripheral Treg cell generation. *Nature*. 2020;581(7809):475–479.
19. Hang S, et al. Bile acid metabolites control TH17 and Treg cell differentiation. *Nature*. 2019;576(7785):143–148.
20. Paik D, et al. Human gut bacteria produce TH17-modulating bile acid metabolites. *Nature*. 2022;603(7903):907–912.
21. Miyamoto Y, et al. Periportal macrophages protect against commensal-driven liver inflammation. *Nature*. 2024;629(8013):901–909.
